# Genomic regions of Solanum tuberosum L. associated with the tuber eye depth

**DOI:** 10.18699/VJ20.638

**Published:** 2020-08

**Authors:** I.V. Totsky, I.V. Rozanova, A.D. Safonova, A.S. Batov, Yu.A. Gureeva, A.V. Kochetov, E.K. Khlestkina

**Affiliations:** Institute of Cytology and Genetics of Siberian Branch of the Russian Academy of Sciences, Novosibirsk, Russia Siberian Research Institute of Plant Production and Breeding – Branch of the Institute of Cytology and Genetics of Siberian Branch of the Russian Academy of Sciences, Novosibirsk, Russia; Institute of Cytology and Genetics of Siberian Branch of the Russian Academy of Sciences, Novosibirsk, Russia Federal Research Center the N.I. Vavilov All-Russian Institute of Plant Genetic Resources (VIR), St. Petersburg, Russia; Siberian Research Institute of Plant Production and Breeding – Branch of the Institute of Cytology and Genetics of Siberian Branch of the Russian Academy of Sciences, Novosibirsk, Russia; Siberian Research Institute of Plant Production and Breeding – Branch of the Institute of Cytology and Genetics of Siberian Branch of the Russian Academy of Sciences, Novosibirsk, Russia; Siberian Research Institute of Plant Production and Breeding – Branch of the Institute of Cytology and Genetics of Siberian Branch of the Russian Academy of Sciences, Novosibirsk, Russia; Institute of Cytology and Genetics of Siberian Branch of the Russian Academy of Sciences, Novosibirsk, Russia; Institute of Cytology and Genetics of Siberian Branch of the Russian Academy of Sciences, Novosibirsk, Russia

**Keywords:** GWAS, SNP, potato, eye depth, GWAS, SNP, картофель, глубина залегания глазков

## Abstract

Potato (Solanum tuberosum L.) is one of the most important food crops in the world. The genome of this potato species is autotetraploid and has a high level of heterozygosity, also this potato species is a cross-pollinated plant. These characteristics complicate the genetic analysis and breeding process. The tuber’s eye depth is an important trait that affects the suitability of potato varieties for processing. Potato breeding for this trait is based on phenotypic assessment. Identification of the loci that control tuber eye depth would allow diagnostic markers for the marker-assisted selection to be created. The aim of this study is to search for loci associated with the eye depth by analyzing Solanum tuberosum varieties from the GenAgro collection of the Institute of Cytology and Genetics of the Siberian Branch of the Russian Academy of Sciences, genotyped using the Illumina 22K SNP potato array DNA chip. The 24 significant markers associated with the “eye depth” trait were identified using 15,214 SNP markers genotyped with the Illumina 22K SNP potato array chip and the general linear model (GLM) taking into account the population structure. Data obtained showed the presence of SNPs in four genomic regions: on chromosome 4 (1 marker in the 3.92 Mb area), 5 (1 marker in the 4.67 Mb area) and 10 (1 marker in the 4.87 Mb area and 21 markers in the region between 48.1–48.9 Mb). The results of localization in the region 48.1–48.9 Mb of chromosome 10 correspond to previously published studies, the remaining three regions were detected for the first time. DNA sections containing SNPs linked to the tuber’s eye depth were studied in the SolTub_3.0 potato genome assembly (https://plants.ensembl.org/). KASP markers were developed based on the data obtained. It will be possible to screen the breeding material and to breed the varieties more effectively using current markers associated with a shallow tuber’s eye depth.

## Abstract (ru)

Картофель (Solanum tuberosum L.) – одна из важнейших в мире продовольственных культур. Геном вида автотетраплоидный, отличается высоким уровнем гетерозиготности, этот вид является также перекрестноопыляемым. Все это затрудняет генетический анализ и селекционный процесс. Глубина залегания глазков клубня картофеля продовольственного назначения – важный признак, влияющий на пригодность сортов картофеля для переработки. Селекция по этому признаку ведется на основе фенотипической оценки. Идентификация локусов, контролирующих данный признак, позволила бы проводить маркер-контролируемый отбор гибридов, отбраковывая формы с глубоким залеганием глазков на ранних этапах селекции. Целью настоящего исследования было выявление геномных районов, ассоциированных с глубиной залегания глазков, путем анализа сортообразцов картофеля S. tuberosum L. из коллекции ГенАгро Института цитологии и генетики СО РАН. При использовании 15 214 SNP-маркеров, генотипированных с помощью чипа Illumina 22K SNP potato array, и обобщенной линейной модели (General Linear Model, GLM) с учетом популя- ционной структуры найдены 24 значимых маркера, ассоциированных с признаком «глубина залегания глаз- ков». Полученные данные показали наличие SNP в четырех геномных районах: в хромосомах 4 (1 маркер в районе 3.92 Mб), 5 (1 маркер в районе 4.67 Mб) и 10 (1 маркер, относящийся к району 4.87 Mб, и 21 мар- кер в районе 48.1–48.9 Mб). Сопоставление выявленных геномных районов в нашем исследовании с более ранними работами подтвердило, что локус между 48.1–48.9 Mб был известен ранее, остальные три района обнаружены впервые. Участки ДНК, содержащие SNP, сцепленные с глубиной залегания глазков, были изучены в сборке генома картофеля SolTub_3.0 (https://plants.ensembl.org/), и на основе полученных данных были разработаны КASP-маркеры, при применении которых можно будет более эффективно вести скрининг селекционного материала и селекцию сортов с мелким залеганием глазков.

## Introduction

Potato Solanum tuberosum L. is the important food crop. The genetic analysis and breeding process of this crop is complicated by the autotetraploid nature and a high level of heterozygosity of this species. In addition, S. tuberosum is a cross-pollinated species, that also makes genetic research and breeding more difficult. (Prashar et al., 2014). Potato varieties are reproduced vegetatively in view of the low fertility and the impossibility of ripening fruits in the climatic conditions of many countries. This type of reproduction allows this crop to maintain the identity of variety genome in different reproduction, despite the heterozygosity.

Mapping of quantitative trait loci (QTL) and genes of autotetraploid potato using biparental populations is a difficult task. The biparental populations method for authotetraploids requires obtaining and analysis of numerous progeny. However, the low fertility of most potato varieties does not allow obtaining big mapping populations. Researches often overcome these limitations of mapping studies by transition to diploid level, at which in addition interspecific hybridization is possible. However this approach still requires high level of fertility. All these features limit the use of S. tuberosum varieties in genetic mapping and QTL studies. However, the possibility of applying a genome-wide association studies (GWAS) with the development of high performance (HP) sequencing and other HP-genotyping methods (including application of SNP-arrays) made it possible to intensify research aimed on identification of genomic loci related with quantitative traits, avoiding all difficulties mentioned above for biparental mapping and QTL analysis. The vegetative reproduction way of cultivated forms of potato facilitates GWAS method compared to other cross-pollinated plants, however some adaptation of the method is still needed taking into account the autotherapoid nature and heterozygosity (Prashar et al., 2014; Khlestkin et al., 2019).

The depth of the tuber eyes is an important trait for the suitability of potato varieties for processing. The volume of losses during peeling, which should not be higher than 15 % (Zemtcova, Timofeeva, 2011), depends on the eye depth. Accordingly, this trait affects the cost of peeling during processing. Based on the conditions for obtaining minimal waste during mechanized abrasive peeling, the tuber eye depth should be no more than 1.5 mm (Pshechenkov, Mal’cev, 2011). Evaluation of potato tuber eye depth is carried out using various scales. A number of studies used scales divided into three to nine grades (Li et al., 2005; Prashar et al., 2014; Hara-Skrzypiec et al., 2018).

The first genetic studies of tuber eye depth were performed at the beginning of the twentieth century. R.N. Salaman (1911) showed that deep eyes dominate shallow ones. Later W. Black (1930) also suggested that the eye depth is controlled by genetic factors, but he hypothesized that this trait has an intermediate type of inheritance or incomplete dominance when extreme phenotypes (very deep and very shallow eyes) are probably homozygous and intermediate phenotypes (medium depth of the eye) are heterozygous. B. Maris (1966) suggested that the eye depth is controlled by one major gene with a additive effect. Some authors have reported that shallow eyes are dominant (Howard, 1974). H. Kukimura (1972) and H.W. Howard (1974) indicated that when crossing two samples with shallow eyes, in segregation can appear samples with deep and medium eyes. Studies that using genetic markers have shown existence of the major locus that controls the eye depth trait (Li et al., 2005). These studies have also shown that the deep eye (Eyd) dominates the shallow eye (eyd). The Eyd/eyd locus, which is responsible for the depth of the eye, is located on chromosome 10 (Li et al., 2005).

W. Black (1930) was first who suggested eye depth to be quite dependent on environmental conditions. Later, B. Maris (1966) demonstrated a high susceptibility of this trait to changes under the influence of environmental factors. However, H.W. Howard (1974) argued that the eye depth is determined mainly by the genotype. Later, a number of studies also showed a high heritability of the eye depth (Gopal et al., 1992; Love et al., 1997; da Silva et al., 2014; Ney et al., 2016) and an insignificant environmental influence (Love et al., 1997). Other authors show that the genotype most strongly affects the trait, but the influence of environmental factors is also quite high (Hara-Skrzypiec et al., 2018). A number of studies have shown the ability of the eye depth to change under the influence of somaclonal variation (Evans et al., 1986; Thieme, Griess, 2005).

In traditional breeding, the depth of the eyes is estimated in the first generation after crossing. However, the tubers of hybrids grown from botanical seeds are too small in size and in this case it is difficult to adequately estimate the depth of the eyes. To solve this problem, DNA markers can be used that will allow effective selection at the first generation stage and reduce the volume of subsequent studies.

The aim of this study was to search for loci associated with the eye depth by analyzing S. tuberosum potato varieties from the GenAgro collection of the Institute of Cytology and Genetics SB RAS, genotyped using the Illumina 22K SNP potato array.

## Materials and methods

**The plant material.** A total of 88 potato varieties from the collection of the GenAgro Institute of Cytology and Genetics SB RAS (Table 1) were phenotyped. Most of the potato collection was represented by varieties and hybrids of domestic selection, some varieties studied were from abroad (Ukraine, Germany, China, etc.).

**Table 1.**
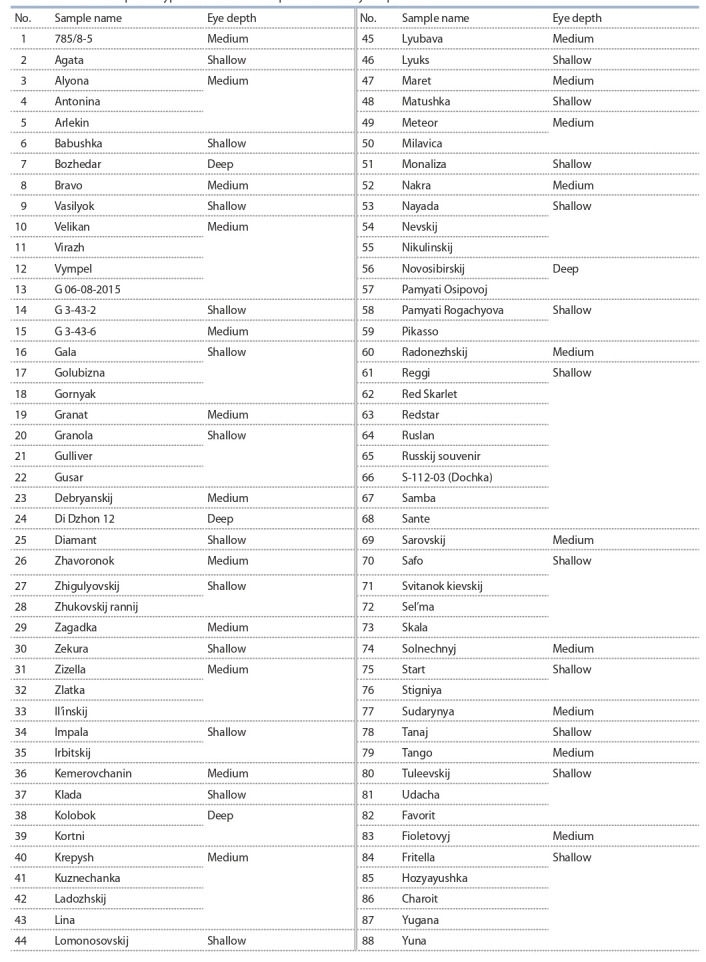
The results of phenotypic estimation of the potato tubers eye depth

**Plants were grown** in the field in two field plots on the territory of the Michurinsky village, Novosibirsk region from May to August 2017.

Field tests were carried out according to the following scheme: the number of rows for each genotype was 2; number of plants in a row – 10; row length – 3 m; the distance between the rows – 0.75 m; the distance between the plants in the rows – 0.30 m; planting method – manually (by hand) on furrows, filling furrows with harrows; landing date is the third decade of May.

Agrochemical characteristics of the soil: the content of exchanged potassium 110.00 mg/kg; the amount of exchanged bases 24.19 mg-eq/100 g; hydrolytic acidity 3.23 mg-eq/100 g; exchanged acidity 5.60 mg-eq/100 g; humus content 2.67 %; the content of mobile phosphorus 5.14 mg/kg; the degree of saturation with bases (V) 88.20 %.

**Meteorological conditions of the growing season:**

May. Air temperature: long-term average 10.90 °C; average monthly 12.60 °C; effective temperature sum 197.60 °C. Precipitation: long-term average 37.00 mm; the amount for the month is 33.90 mm.

June. Air temperature: long-term average 16.90 °C; average monthly 19.30 °C; effective temperature sum 576.00 °C. Precipitation: long-term average 55.00 mm; the amount for the month is 71.90 mm.

July. Air temperature: long-term average 19.40 °C; average monthly 18.50 °C; effective temperature sum 1004.00 °C. Precipitation: long-term average 61.00 mm; the amount for the month is 99.50 mm.

August. Air temperature: long-term average 16.20 °C; average monthly 16.80 °C; effective temperature sum 1408.00 °C. Precipitation: long-term average 67.00 mm; the amount for the month is 65.60 mm.

**Evaluation of the eye depth** was carried out in 2017 in accordance with the VIR methodology (Kiru et al., 2010). The eye depth was determined on a scale of 1 to 3: 1 – shallow (less than 1–1.3 mm), 2 – medium (1.4–1.6 mm), 3 – deep (more than 1.7 mm). Five typical eyes of a potato tuber were measured. These data were compared with the data presented in the database of the State Register of breeding achievements approved for use (http://gossortrf.ru/gosreestr.html) and data from the European cultivated potato database (https://www.europotato.org/).

**For genotyping,** DNA was isolated from the skin of potato tubers using the DNeasy Plant Mini kit (Qiagen, CA, USA) according to the manufacturer’s protocol. The concentration and purity of the test samples was determined using gel electrophoresis and using a Nanodrop 2000 apparatus.

All 88 varieties were genotyped using the Illumina 22K SNP potato array (GGP Potato V3) DNA chip at Traitgenetics GmbH (Gatersleben, Germany).

The 21,226 SNP dataset was filtered using Excel software. Low quality data was deleted, all monomorphic markers and markers containing more than 95 % of one allele. For further analysis, 15,214 (71.7 %) SNPs were taken. The chromosomal position for SNP has been determined using data of (Vos et al., 2015).

**An analysis of the associations** between the eye depth and the genomic regions was carried out using the Tassel 5.2.59 software package (Bradbury, 2007). A generalized linear model (GLM) was used taking into account the population structure (Q). The population structure of the collection was analyzed in previous works (Khlestkin et al., 2019) using the STRUCTURE v 2.3.4 program (Pritchard et al., 2000), based on data from all 15,214 markers taken for further analysis.

Since the TASSEL software package was developed for the analysis of the genomes of diploid species, for its application to the tetraploid genome, the data were transcoded into a digital format taking into account the representation of the effector allele (Khlestkin et al., 2019).

Two criteria were used to determine the significance of associations: (1) the Bonferroni correction, which was defined as dividing the statistical significance level (0.05) by the total number of trials, in our case, by the number of markers (15,214) and amounted to 3.28·10^–6^, and (2) Benjamini– Hochberg test (Benjamini, Hochberg, 1995) (false discovery rate, FDR). In this case, only those SNPs whose p-value (FDR) did not exceed the threshold value of 0.05 were considered significant, taking into account the amendments to the Benjamini–Hochberg method.

**The development of KASP markers** and genotyping of 86 varieties using them was carried out by LGC Genomics LLC (Teddington, UK). Sections of 100 bp DNA containing SNP associated with the eye depth trait were converted to KASP markers (Supplementary 1)^1^. Data about the nucleotide composition of these sites are presented in the SolTub_3.0 potato genome assembly (https://plants.ensembl.org/). In the future, it will be possible more effectively carry out of screening breeding material and selection varieties with a shallow eye depth using these developed KASP markers.

Supplementary Materials are available in: https://vavilov.elpub.ru/jour/manager/files/SupplTotsky_engl.pdf



## Results

**Phenotyping** conducted on 88 samples of the collection showed that most of the collection, 49 samples, had a shallow eye depth. Also, a large part of the collection was represented by samples having a medium eye depth – these were 33 genotypes. Samples with deep eyes represented a small part of the sample and were represented by only 6 samples (see Table 1).

Association studies using GLM and taking into account the population structure revealed 24 SNPs significantly associated with the tuber’s eye depth (Table 2, see the Figure). After applying the Bonferroni multiple test correction at 5 % ( p < 3.28·10^–6^), only 15 of 24 SNPs remained significant for the tuber’s eye depth, the remaining nine SNPs remained significant only using the FDR criterion ( p < 0.05).

**Fig. 1.**
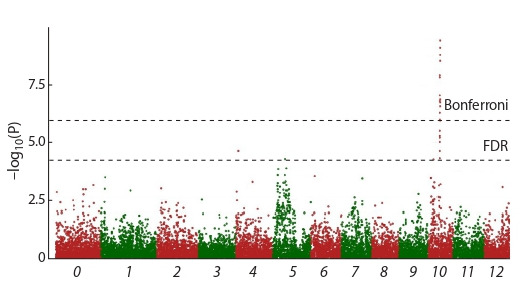
Manhattan plot showing significant SNP, associated with eye depth when using GLM analysis taking into account the population structure. 1–12 – chromosome designation; 0 – SNPs unassigned to certain chromosomes.

**Table 2.**
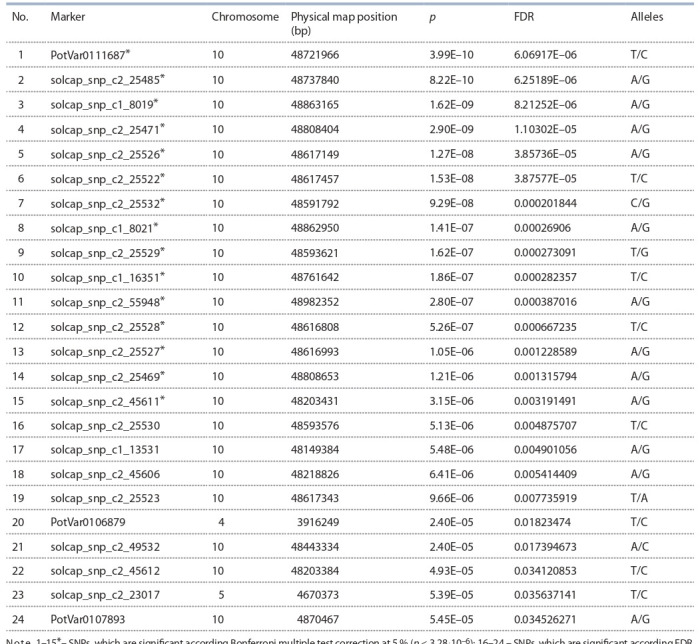
SNPs associated with the eye depth, which identified using GLM analysis Note. 1–15*– SNPs, which are significant according Bonferroni multiple test correction at 5 % ( p < 3.28·10–6); 16–24 – SNPs, which are significant according FDR.

SNPs associated with the eye depth are found in four genomic regions, two of which are located on chromosome 10. SNPs that are significant when using Bonferroni’s multiple test corrections ( p < 3.28·10–6) were located on one of the two loci of chromosome 10. SNPs that are significant when using FDR ( p < 0.05) are found at all four loci on chromosomes 10, 4, and 5. On chromosomes 4 and 5, only one significant SNP was located. Twenty-one SNPs were located on chromosome 10 in a relatively small area between 48.1 and 48.9 Mb. Another SNP was located in another region of chromosome 10 at position 4.87 Mb (this SNP is significant when using FDR).

The most significant SNP PotVar0111687 refers to a noncoding sequence. In total, among 18 significant SNPs, 6 are located in non-coding regions of the genome, and 11 are located in protein coding sequences. Annotation of genes containing significant SNPs is given in Supplementary 2. Due to the lack of accurate information on the genetic control of the eye depth, it turned out to be difficult to definitely relate the identified genes to the formation of the eye depth.

## KASP genotyping analysis results

**KASP genotyping** was carried out using 11 KASP markers developed on the basis of SNP that significant when using Bonferroni multiple test corrections, which were linked to the eye depth and located on the 10th chromosome between 48.1 and 48.9 Mb (Table 3). Eight markers correlated with the eye depth, or rather, the homozygous state of one of the alleles of these markers correlates with a shallow eye.

**Table 3.**
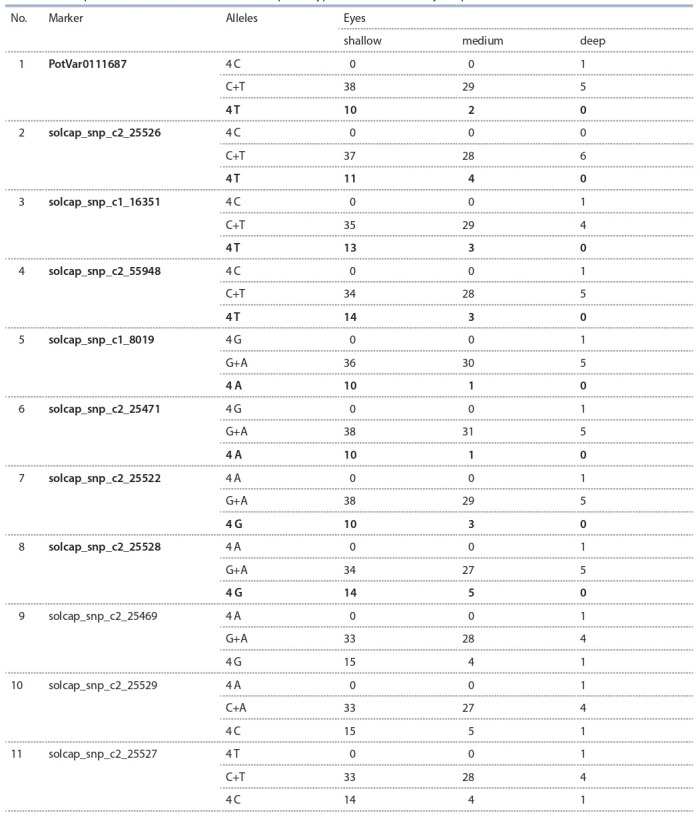
Correspondence of the allele of marker and phenotypic condition of the eye depth Note. Haplotypes predicting small eyes depth are highlighted in bold.

Haplotypes that are 100 % predictive for shallow eyes are: PotVar0111687 (homozygote for T), solcap_snp_ c1_16351 (homozygote for T), solcap_snp_c2_55948 (homozygote_s2, 55848), solcap_snp_c2_25522 (homozygous for G), solcap_snp_c2_25528 (homozygous for G). The carriers of this haplotype are the Vasilyok, Gulliver, Lyuks, Matushka, Nayada, Dochka, Tuleevskij, Favorit, Charoit varieties.

## Discussion

X.Q. Li et al. (2005) conducted genetic mapping of the locus responsible for the eye depth. As a result, the Eyd locus was mapped on chromosome 10 proximal to the CT240 marker (12 cM) and distal to the STM0051 marker (13 cM). BLAST analysis of the sequences of these markers indicates the position of CT240 in the region of 51 Mb, and STM0051 in the region of 23.4 Mb. It can be assumed that the locus that we identified on chromosome 10, which is located between 48.1 and 48.9 Mb, corresponds to the Eyd gene. In a number of works (Śliwka et al., 2008; Prashar et al., 2014; Rosyara et al., 2016 and others), a locus linked to eye depth was regularly detected in this region.

A. Prashar et al. (2014) using Infinium 8303 Potato Array compiled a genetic map of diploid potatoes. After selecting the most valuable SNPs, the map contained 1355 different loci and 2157 SNPs. In this work, it was found that the main locus for the tuber’s eye depth is located on chromosome 10 (SNP: c1_8020) and linked to the tuber-shaped locus. This SNP is located on chromosome 10 in the region of 48.8 Mb, as well as locus 4 detected in the current study. Also A. Prashar et al. (2014) found two SNPs linked to the eye depth on chromosomes 2 (c2_7422) and 3 (c2_37119), which were not so significant and most likely could have an auxiliary effect.

U.R. Rosyara et al. (2016), also used GWAS to search for SNPs linked to the eye depth. In their study, the DNA chip had 3.5 thousand markers. A highly significant SNP for the eye depth was located at the 48.9 Mb position on chromosome 10 (coinciding with the locus detected by A. Prashar et al. (2010) and locus 4, identified in our work). The less significant SNP c2_11685, which was also linked to the tuber’s eye depth, was located on chromosome 5 at 2.3 Mb position. In our work, we also detected SNP associated with the eye depth, located on chromosome 5, but in a distinct region – 4.7 Mb.

H. Lindqvist-Kreuze et al. (2015) also found potato tubers eye depth QTL on chromosome 10 at position 49.4 Mb, as well as another locus on chromosome 12. Researchers have identified a number of candidate genes that underlie significant QTL and are linked to the eye depth. One of them is the BEL-1-like homeobox gene, found at a distance of 1.37 Mb from the QTL marker toPt-437059 on chromosome 10. The second is the α-expansion gene, which was found in the region of significant QTL on chromosome 10 at a distance of 1.78 Mb from the QTL marker toPt-437059. Also, some genes associated with the production and modification of pectins were found in the close proximity to toPt-437059 marker on chromosome 10.

A. Hara-Skrzypiec et al. (2018) also performed mapping for a number of potato traits, including the eye depth. Seven QTLs that were linked to the eye depth were found: one per each chromosome 1, 4, and 11, and two per each chromosomes 3 and 5. However, unlike other studies, the major QTL was located on chromosome 4 (at position 68.8 Mb) and accounted for 22.6 % of the dispersion in the average data set. We found in our study a significant SNP PotVar0106879 located on chromosome 4 at position 3.9 Mb.

Thus, the key locus responsible for the eye depth is located on chromosome 10, while loci with a minor effect are located on chromosomes 1, 2, 3, 4, 5, 11, and 12, as well as in other parts of chromosome 10.

## Conclusion

Despite a wide range of genetic studies of the potato tubers eye depth trait and the identification of genes and QTL associated with eye depth variability, DNA markers are still not used in the breeding of potatoes to this trait. Meanwhile, the use of diagnostic DNA markers allows to carry out more efficient pre-breeding research (screening of potato genetic resources to identify donors of valuable allelic variants) and marker-assisted selection in breeding programs (Gebhardt et al., 2006; Chen et al., 2017; Klimenko et al., 2017, 2019). We were able to develop a number of new PCR markers that can be convenient for screening the genetic resources and breeding material of potato. After additional verification of these proposed markers on an extended sample, they can be used to select shallow-eyed plants by analysis at the DNA level. We selected PCR markers located on chromosome 10 at positions between 48.62 and 48.98 Mb. These data coincide with the data of other authors on the location of the supposed gene, which is responsible for the eye depth. However, due to the larger number of markers that we found, these data allow us to clarify the localization of the gene of interest to us and suggest that it is located at positions between 48.0 and 49.0 Mb. According to our data, selection according to the haplotype which includes 8 SNPs located on chromosome 10 is optimal (PotVar0111687 (homozygote of T), solcap_snp_c2_25526 (homozygote of T), solcap_snp_c1_16351 (homozygote of T), solcap_ snp_c2_55948 (homozygote of T), solcap_snp_c1_8019 (homozygote of A), solcap_snp_c2_25471 (homozygote of A), solcap_snp_c2_25522 (homozygote of G), solcap_ snp_c2_25528 (homozygous for G)). The carriers of this haplotype are the varieties Vasilyok, Gulliver, Lyuks, Matushka, Nayada, Dochka, Tuleevskij, Favorit, Charoit, which are characterized by a shallow eye depth. The use of these varieties as donors of this trait (which are at the same time donors a number of other valuable properties) in combination with PCR analysis of the offspring to select carriers of the corresponding haplotype will provide a more economical and accelerated method of creating potatoes with a shallow tuber eye depth.

## Conflict of interest

The authors declare no conflict of interest.
